# Reporting Two Novel Mutations in Two Iranian Families with Cystic Fibrosis, Molecular and Bioinformatic Analysis

**DOI:** 10.52547/ibj.3713

**Published:** 2022-11-08

**Authors:** Amin Hosseini Nami, Mahboubeh Kabiri, Sirous Zeinali

**Affiliations:** 1 Department of Biotechnology, College of Sciences, University of Tehran, Tehran, Iran;; 2 Dr. Zeinali’s Medical Genetics Laboratory, Kawsar Human Genetics Research Center, Tehran, Iran

**Keywords:** Cystic fibrosis, Cystic fibrosis transmembrane conductance regulator, Genetic linkage, Haplotype, Sequence analysis

## Abstract

**Background:**

Cystic fibrosis is the most common heredity disease among the Caucasian population. More than 350 known pathogenic variations in the *CFTR* gene (NM_000492.4) cause CF. Herein, we report the outcome of our investigation in two unrelated Iranian families with CF patients.

**Methods:**

We conducted phenotypic examination, segregation, linkage analysis, and *CFTR* gene sequencing to define causative mutations.

**Results:**

We found two novel mutations in the present study. The first one was a deletion causing frameshift, c.299delT p.(Leu100Profs*7), and the second one was a missense mutation, c.1857G>T, at nucleotide binding domain 1 of the CFTR protein. Haplotype segregation data supported our new mutation findings.

**Conclusion:**

Findings of this study expand the spectrum of *CFTR* pathogenic variations and can improve prenatal diagnosis and genetic counseling for CF.

## INTRODUCTION

Cystic fibrosis (OMIM: #219700), a congenital disease with an autosomal recessive mode of inheritance, is caused by mutations in the *CFTR* gene (OMIM: *602421; cytogenetic location: 7q31.2)^[^^1^^]^. These mutations affect the function of the CFTR protein in ion channels in epithelial tissues, leading to unusually viscous secretions. This abnormality gives rise to obstruction in lung airways and pancreatic ducts. Individuals with *CFTR* mutations have shown susceptibility to bacterial infections^[^^2^^,^^3^^]^. 

CF is the most frequent fatal autosomal recessive heredity disease among the Caucasian population with an average incidence of 1 out of 3,500 individuals in Europe^[^^4^^]^. Besides, one in every 2,500, 3,600, and 4,000 children in Australia, Canada, and the US are respectively born with CF^[^^5^^]^. So far, CF Mutation Database has reported more than 2,000 *CFTR* gene variations, of which only 352 have been verified to be pathogenic. 

New advances in genetic technology and availability of powerful predictive tools have accelerated the findings of disease-causing mutations, including alterations in the *CFTR* gene in CF patients and carriers^[^^6^^]^. Furthermore, the discovery of novel variants supplements the information about the spectrum of the *CFTR* mutations. These findings are essential for geneticists and clinicians working on CF diagnosis, prevention, and treatment, as well as for those seeking for new therapeutic approaches. Currently, there are powerful tools for the identification and characterization of newly discovered mutations or variants. Both *in silico* and molecular findings may be necessary to verify a mutation as pathogenic or nonpathogenic. 

Consanguineous marriage plays a crucial role in the relatively high incidence of CF in Iran, as observed in several other autosomal recessive disorders^[^^7^^-^^11^^]^. While CF is believed to be rare in Iran, an earlier investigation has suggested that it might be an underdiagnosed disorder in the country^[^^12^^]^. 

The present study aimed to investigate nine individuals from two unrelated families who had affected children with CF. To this end, we performed phenotypic examination, pedigree study, and genetic analysis by Sanger sequencing and haplotyping using the *CFTR*-linked STR markers.

## MATERIALS AND METHODS

Subjects

Two Iranian families with children suspected of being affected with CF were referred to Dr. Zeinali’s Medical Genetic Lab., Kawsar Human Genetics Research Center (KHGRC) for *CFTR* gene analysis. Each family had four members. The affected child who belonged to the family I was a four-month-old male infant at the time of counseling. The affected child from family II was a female infant who had died at two months of age. Peripheral blood samples were collected in EDTA-containing tubes.

DNA extraction and genotyping

DNA samples were extracted by salting-out method^[^^13^^]^. The concentration of the isolated DNA was measured by Nanodrop spectrophotometry (Thermo Fisher Scientific, Foster City, CA, USA). Genetic analysis of the DNA samples was performed using direct sequencing of the *CFTR* gene exons. Primers for sequencing were designed to target all exons, and 200 flanking intronic regions were used based on a previously reported method^[^^14^^]^. Sequences of primers are available upon request. DNA sequencing was carried out using BigDye Terminator Cycle Sequencing Kit (Thermo Fisher Scientific) and analyzed on 3130/XL Genetic Analyzer. By using bioinformatics tools such as MutationTaster^[^^15^^]^, PolyPhen-2^[^^16^^]^, CADD^[^^17^^]^, FATHMM^[^^18^^]^, SIFT^[^^19^^]^, and PROVEAN^[^^20^^]^, we investigated the pathogenicity of the detected variations, including novel variants^[^^21^^]^. Mutation nomenclature was compiled in accordance with the Human Genome Variation Society guidelines^[^^22^^]^. Novelty and pathogenicity of the mutations were also investigated in the Human Gene Mutation (http://www. hgmd.cf.ac.uk/ac/all.php), Clinical and Functional Translation of CFTR (http://cftr2.org), and CF mutation (http://www.genet. sickkids.on.ca) databases, and also in literature review. The protein tertiary structure was predicted by Swiss-MODEL software^[^^23^^,^^24^^]^.

Short tandem repeat-based homozygosity mapping

We examined the pattern of inheritance by CFTR-linked STR markers using GT Hapscreen CFTR kit (Genetek Biopharma, Berlin, Germany). As per the kit user manual, we drew and interpreted each person’s haplotype. We also performed multiplex PCR using the GT Hapscreen CFTR kit, and the fragments were analyzed on the ABI 3130/XL Genetic Analyzer. The resulting files were converted to PDF using GeneMapper IDX 1.5, and fragment sizes were used to draw haplotypes according to the manufacturer’s user manual.

## RESULTS

Clinical presentation

Family I

**Fig. 1:**
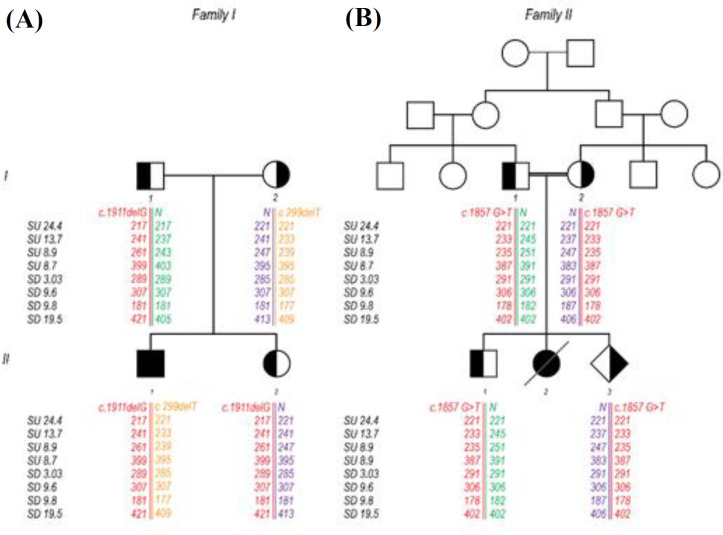
Pedigree of two families with novel CFTR variants. (A) In family I, the I1, II1, and II2 share the same haplotype and mutation. The II1 shares a similar haplotype with I2, as well. Therefore, the defective haplotypes in the II2 have come from the father even if we do not show the mutation. (B) In family II, I1 and I2 are cousins and share the same mutation and haplotype. Their affected child (II2) has died, and the other two children are carriers based on mutation and haplotype results. II1 has received the defective haplotype from the mother and II3 from the father

The family had been referred to Kawsar Human Genetics Research Center (KHGRC) for prenatal diagnosis. The parents were not consanguineous (Fig. 1A). The proband’s (II-1) sweat test was positive for CF. Also, the proband manifested classic CF-related symptoms, i.e. salty-tasting skin from birth and greasy stools. The elastase activity in his stool was severely insufficient (50 µg/g), and microscopic analysis of the stool had revealed many fatty acid droplets. At the time of our examination, the infant had already surgical treatment for ileal atresia. The infant’s father, mother, and sister displayed no sign of CF. Haplotyping and mutation analysis of proband’s sister (II-2) indicated that she is carrier of mutation inherited from her father. 

Family II

A consanguineous couple of Kurdish origin (Fig. 1B) was referred to HGRC for prenatal diagnosis. The mother, a 29-year-old woman, was at 12 weeks of gestation at the time of blood sampling. The deceased female child (i.e., II2, Fig. 1B) had been affected with CF as the positive sweat test confirmed the diagnosis. The family had an 11-year-old son with no CF-associated complications. He also participated in this study. 

Sanger sequencing and identification of two novel variants in the *CFTR* gene

The analysis of sequencing revealed three mutations in the studied participants. In family I, we identified two mutations that one of them was a previously described pathogenic deletion, c.1911delG^[^^25^^]^ p.(Gln637Hisfs *26), in exon 13 of the proband’s sample. His father and sister were heterozygous for this mutation. Another heterozygote mutation, c.299delT p.(Leu100 Profs*7), was detected in exon 4 of the proband. This mutation shared by his mother confirmed to be a novel mutation associated with the patient's phenotype, since we did not find any record of c.299delT mutation in the CF Mutation Databases or the literature. Therefore, this mutation can be regarded as a novel genetic variation.

**Fig. 2:**
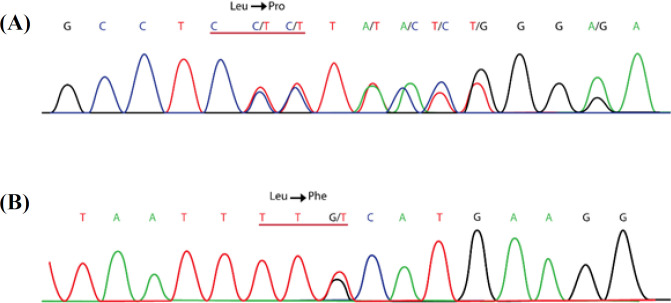
Result of Sanger sequencing of studied probands. The CFTR variant of (A) c.299delT p.(Leu100Profs*7) and (B) c.1857G>T p.L619F were found in family I and II, respectively

**Fig. 3:**
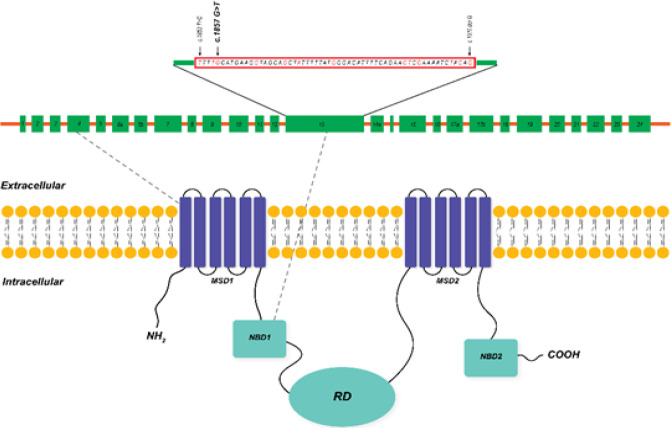
Exon structure of the CFTR gene and domain structure of the CFTR protein. The mutation hot spot in exon 13. The mutation point is indicated with red line. The position of variants c.299delT p.(Leu100Profs*7) and c.1857G>T (p.L619F) is shown in the three-dimensional structure of CFTR protein. RD, regulatory domain

**Fig. 4:**
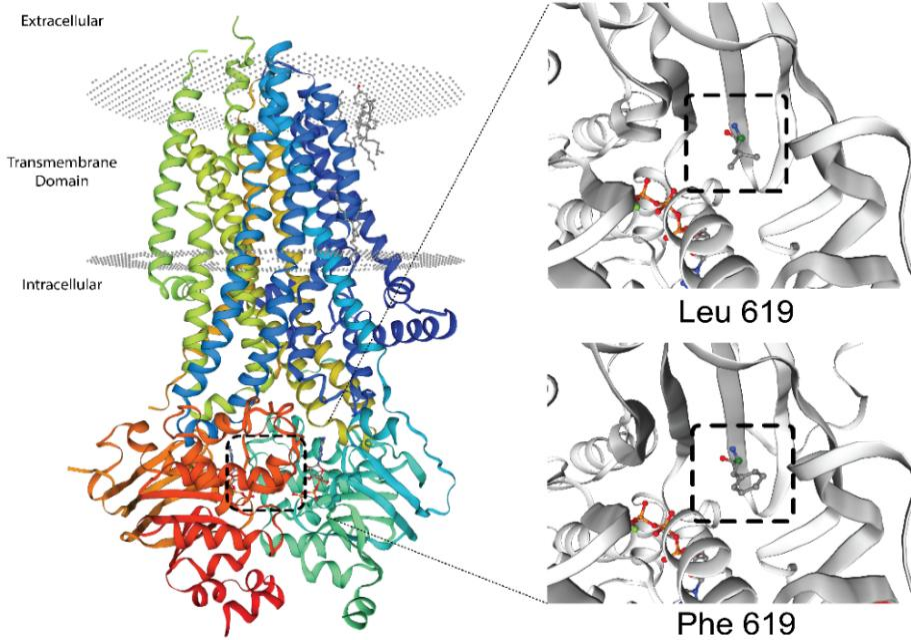
The CFTR protein tertiary structure predicted by Swiss-MODEL software. Different structures caused by amino acid changes are shown in position 619 of CFTR. On the right top the wild-type leucine and on the right bottom, the mutant phenylalanines are shown

The T deletion at nucleotide 299 (Fig. 2A) causes a frameshift and changes the amino acids (aa) frames, p.(Leu100Profs*7). The frameshift caused by this deletion led to substituting the isoleucine codon with a stop codon at aa 106 (p. I106*). MutationTaster predicted this variant as deleterious. No other mutation in other exons merited the same criteria. All pedigree members in family II, who participated in the study, carried a heterozygous mutation c.1857G>T (Fig. 2B), located in exon 13. This missense mutation caused the substitution of leucine to phenylalanine at position 619 (p.Leu619Phe). There is no previous report on c.1857G>T mutation in the CF Mutation Databases and literature; therefore, it is novel. The *in silico* tools predicted this mutation to be damaging and disease-causing, and its CADD score was 23.0. This mutation is in the NBD1 of the CFTR (Fig. 3). Also, *CFTR*-linked STR markers showed that the parents shared the same haplotype and none of the healthy members, including the carrier infant, was homozygote for this haplotype (Fig. 1B). Thus, the segregation analysis of the STR markers suggests an association of c.1857G>T mutation with CF phenotypes in the deceased infant. The Swiss-MODEL predicted the three-dimensional structure of the CFTR protein (Fig. 4). This amino acid substitution in position 619 might affect the stability of the protein.

## DISCUSSION

By investigating molecular defects causing CF in our patients, we found two novel mutations, c.299delT and c.1856G>T, in two unrelated families. There is no previous report on these two mutations in the CFTR databases, Human Gene Mutation Database, and literature; thus, they can be regarded as novel mutations. Results from this study expand the mutation spectrum of CF disease and will be of great help for prenatal diagnosis and carrier detection of this disorder worldwide.

**Fig. 5:**
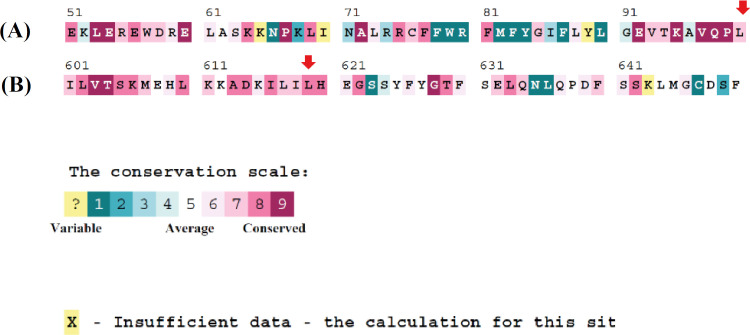
Conservation score of CFTR protein by ConSurf software. Conservation score of the amino acids (A) 51-100 and (B) 601-650 (B); red arrows indicate the position of 100^th^ and 619^th^ amino acid, respectively

The affected child in our first case (family I) had typical CF symptoms and was compound heterozygote for c.299delT p.(Leu100Profs*7), and c.1911delG (p.Gln637Hisfs*26) mutations. The novel p.(Leu100Profs*7) mutation was located in the first membrane spanning domain of the CFTR protein and a

*CFTR* gene mutation hotspot^[^^26^^,^^27^^]^ (Fig. 3). The mutation causes a six-amino-acid alteration, and a termination codon follows the frameshift caused by this deletion. The nonsense-mediated mRNA decay targets the transcribed mRNA after this premature termination^[^^28^^]^. This process results in the loss of CFTR protein activity and is consistent with the patient's classical CF phenotypes. The c.299delT p.(Leu100 Profs*7) mutation is classified as pathogenic based on the ACMG guidelines because it meets PVS1, PM2, PM3, and PP3 criteria. It has formerly been classified as class I mutation, as well^[^^29^^]^. *In vitro* analysis can verify the effect of this variant in CF. The patient also carried a second mutation, a previously known deletion, the c.1911delG (p.Gln637Hisfs*26) in exon 13. This mutation has been reported in patients with pancreatic insufficiency and pathological lung conditions^[^^30^^,^^31^^]^. The c.1911delG, legacy name c.2043delG, is a common mutation in the north of Iran, where the patients are originally from^[^^31^^]^. Furthermore, several studies have reported this mutation in a number of areas with geographical proximity to Iran, e.g. Russia^[^^32^^,^^33^^]^ and the Middle Eastern countries such as Bahrain^[^^34^^]^, Turkey^[^^35^^,^^36^^]^, Lebanon^[^^37^^]^, and Saudi Arabia^[^^38^^]^. 

We found a second novel missense mutation in another family having c.1857G>T (p.L619F) mutation. This mutation has a length of 58 nucleotides and is positioned in a mutation hot spot in exon 13, which harbors 15 mutations (12 missense mutations and three deletions) as reported in the CF Mutation Database so far. The mutation is also close to another nonsense mutation hotspot region in exon 13. The altered amino acid is placed in the NBD1 of CFTR protein^[^^39^^]^. The NBD1 is a key player in the CFTR gating control because of its interaction with ATP^[^^40^^,^^41^^]^. This mutation introduces an amino acid with different properties, affecting the structural stability and gating function of the protein. Also, the wild-type amino acid, which is leucine and the mutant amino acid, phenylalanine, differs in size. The mutant residue is bigger than the wild type; therefore, its bulky side chain might lead to bumps (Fig. 4). H-loop is a conserved structure^[^^40^^]^ in CFTR protein, which is involved in ATP recognition^[^^42^^]^. The c.1857G>T substitution is located seven amino acids downstream of h-loop. All the *in silico* tools predicted this mutation to be damaging, and based on the ACMG guidelines, this mutation is likely pathogenic as it meets PP1, PP2, PP5, and PP3 criteria. *In vitro* as well as *in vivo* confirmatory studies can assess the pathogenic effect of c. 1857G>T in CF patients.

We may here note another adjacent mutation, c.1856T>C (p.L619S), detected in other studies^[^^43^^-^^45^^]^. This mutation also changes leucine at position 619 and has been found in a patient with pancreatic insufficiency^[^^43^^]^. Furthermore, when c.1856T>C mutation was introduced in mammalian HEK 293 cells. The transformed cells displayed no Cl^-^ channel activity in the electrode voltage clam test. The mutated cells failed to process the CFTR protein correctly; as a result, the protein was mislocalized^[^^44^^]^. Moreover, nine other mutations approximate to this mutation (aa 601-619) led to CFTR protein processing defects^[^^45^^]^. This high number of pathogenic mutations is consistent with the highly conserved amino acid sequences neighboring the aa position 619 between various species (Fig. 5) and highlights the crucial role of this region, particularly the leucine at position 619 in protein function. 

The infant family II had been diagnosed with CF and died very early at two months of age. Although a small population makes it challenging to make a genotype and phenotype connection, haplotyping indicated that c.1857G>T is probably the cause of CF in the infant. Therefore, we can deduce that the mutated allele segregates with a specific haplotype using *CFTR* linked STR markers. 

In the present study, we discovered two novel mutations (c.299delT and c.1857G>T) and another missense mutation, c.1911delG of CF disease. Introducing these two novel mutations to the *CFTR* mutation spectrum will help genetic specialists and clinicians better diagnose CF patients and provide more effective medical care. Applying haplotyping will increase the accuracy of findings, particularly in families with few children or consanguinity.

## DECLARATIONS

### Data availability

The raw data supporting the conclusions of this article are available from the authors upon reasonable request.
